# Coadministration of Lopinavir/Ritonavir and Rifampicin in HIV and Tuberculosis Co-Infected Adults in South Africa

**DOI:** 10.1371/journal.pone.0044793

**Published:** 2012-09-28

**Authors:** Richard A. Murphy, Vincent C. Marconi, Rajesh T. Gandhi, Daniel R. Kuritzkes, Henry Sunpath

**Affiliations:** 1 Medical Unit, Médecins Sans Frontières/Doctors Without Borders, New York, New York, United States of America; 2 Infectious Diseases Division, Department of Medicine, Emory University School of Medicine, Atlanta, Georgia, United States of America; 3 Infectious Diseases Unit, Department of Medicine, Massachusetts General Hospital, Boston, Massachusetts, United States of America; 4 Division of Infectious Diseases, Department of Medicine, Brigham and Women’s Hospital and Harvard Medical School, Boston, Massachusetts, United States of America; 5 Department of Medicine, McCord Hospital and Infectious Diseases Unit, Nelson Mandela School of Medicine, Durban, South Africa; University of Pittsburgh Center for Vaccine Research, United States of America

## Abstract

**Background:**

In HIV-infected patients receiving rifampicin-based treatment for tuberculosis (TB), the dosage of lopinavir/ritonavir (LPV/r) is adjusted to prevent sub-therapeutic lopinavir concentrations. In this setting, South African clinicians were advised to administer super-boosted LPV/r (400 mg/400 mg) twice daily, instead of standard dosed LPV/r (400 mg/100 mg) twice daily. We sought to determine – in routine practice – the tolerability and HIV treatment outcomes associated with super-boosted LPV/r compared to unadjusted LPV/r in combination with rifampicin-based TB treatment.

**Methodology/Principle Findings:**

We conducted a retrospective review of HIV-infected patients who receiving second-line ART with a LPV/r-containing regimen who required concomitant TB treatment. We identified 29 patients; the median age was 36 years (IQR 29–40), 22 (76%) were female, the median CD4 cell count and viral load at first-line ART failure was 86 cells/mm^3^ (IQR 21–159) and 39,457 copies/mL (IQR 6,025–157,500), respectively. According to physician preference, 15 (52%) of 29 patients received super-boosted LPV/r (400 mg/400 mg) every 12 hours during TB treatment and 14 (48%) of 29 patients received standard dose LPV/r (400 mg/100 mg) twice daily during TB treatment. Among patients who received super-boosted LPV/r there was a trend towards a higher rate of symptomatic transaminitis (27% vs. 7%; p = 0.3), gastrointestinal toxicity (20% vs. 0%; p = 0.2) and a significantly increased need for treatment discontinuation (47% vs. 7%; p = 0.035. The durability of coadministered treatment was significantly shorter in patients who received super-boosted lopinavir/ritonavir with TB treatment compared to patients who received standard lopinavir/ritonavir dosing (log rank, *P* = 0.036). The rate of virologic failure was not higher in patients with unadjusted LPV/r dosing.

**Conclusions/Significance:**

We observed a high rate of toxicity and need for treatment discontinuation among patients on standard rifampicin-based TB treatment who received super-boosted LPV/r.

## Introduction

A significant proportion of HIV-infected patients in South Africa require second-line ritonavir-boosted protease inhibitor (PI)-based antiretroviral therapy (ART) as a result of virologic failure or intolerance of initial ART [1]. According to WHO estimates, more than 100,000 patients have initiated second-line ART, most commonly with a lopinavir/ritonavir (LPV/r)-containing regimen [2]. However the management of tuberculosis (TB) coinfection in such patients is challenging because rifampicin – the cornerstone of antituberculous therapy – leads to a substantial reduction in PI concentrations through the induction of cytochrome p450 enzymes [3], [4]. When administered with rifampicin, reductions of greater than 90% in PI trough concentrations have been observed [5], [6].

The reduction in PI concentrations associated with concomitant rifampicin can be attenuated with the use of higher doses of ritonavir [3]. As a result, among patients receiving TB treatment, clinicians in South Africa were advised to administer super-boosted LPV/r (400 mg/400 mg) twice daily, instead of standard dosed LPV/r (400 mg/100 mg) twice daily [7]. However, in a previous study of healthy volunteers, super-boosted LPV/r was associated with a high rate of nausea, vomiting and transaminase elevations, resulting in early study termination [8]. Poor regimen tolerability was also seen when adult patients with HIV and TB were treated with super-boosted LPV/r and rifampicin in the Netherlands, with suboptimal antiviral efficacy observed in coinfected patients who received standard LPV/r dosing [9].

Although widely used, there is very limited data from routine clinical settings in low and middle-income countries on the tolerability and antiviral efficacy of super-boosted lopinavir in HIV-infected adult patients receiving rifampicin for TB infection. We hypothesized that coinfected patients who received super-boosted lopinavir (400 mg twice daily) would experience a significantly higher rate of adverse events and tolerate a shorter duration of concomitant TB treatment compared to patients receiving standard lopinavir dosing.

## Methods

McCord Hospital, a state-aided hospital, provides treatment for HIV and tuberculosis to patients living in Durban and the province of KwaZulu-Natal. We conducted a retrospective review of HIV-infected patients who initiated second-line ART containing LPV/r at McCord Hospital between July 2004 and February 2007. Eligible for inclusion in the current study were adult patients who received both LPV/r-containing second-line ART and rifampicin for tuberculosis treatment for at least three months, and who underwent a viral load test during the overlap period. There was considerably clinician-to clinician variability regarding the decision to use – during treatment of tuberculosis in patients receiving LPV/r-based second-line ART – either super-boosted LPV/r (400 mg/400 mg) twice daily or standard boosted LPV/r (400 mg/100 mg) twice daily. In all instances, the dose of the lopinavir component was 400 mg twice daily and the nucleoside analogue dosing was standard. We excluded women who received LPV/r-containing ART only as part of a prevention of a maternal-to-child-transmission protocol.

Rifampicin was administered as part of a 4-drug (rifampicin, isoniazid, pyrazinamide, ethambutol) fixed-dose combination (FDC) or as a 2-drug (rifampicin and isoniazid) FDC, depending upon whether the patient was receiving intensive phase or continuation phase TB treatment. The second-line ART agents available during the study included lopinavir/ritonavir [LPV/r 400/100 mg; available during the study period as soft-gel formulation (Kaletra)]; ritonavir, lamivudine, didanosine (enteric-coated formulation); zidovudine; and stavudine.

Patient monitoring followed South African Department of Health recommendations including HIV-1 RNA level (detection limit of <50 copies/ml) and CD4 cell count monitoring every 6 months. Liver function testing was not obtained routinely during coadministration of LPV/r and rifampicin-containing TB treatment but was available to clinic staff in the presence of symptoms or signs of hepatitis (defined as unexplained anorexia, nausea, right upper quadrant pain or the presence of clinical jaundice). Adverse events and abnormal laboratory results during coadministration were graded according to the Division of AIDS Regulatory Support Center guidance (7).

Using a standardized instrument, we abstracted from the patient medical record clinical and demographic characteristics including HIV-1 RNA levels, CD4 cell count, tuberculosis treatment history and outcomes, adverse events detected by clinician and treatment modification.

We compared baseline characteristics (at the time of first-line ART failure), rates of key adverse events, treatment discontinuation and virologic suppression among patients receiving second-line ART and concomitant tuberculosis therapy who received super-boosted lopinavir (400 mg twice daily) and patients who received standard boosted lopinavir (100 mg twice daily). Categorical variables were compared using chi-square and Fisher’s exact tests, and continuous variables were compared using Student’s t-test. We defined the event treatment discontinuation as the need to stop either rifampicin-based TB therapy or LPV/r-based ART prematurely resulting from toxicity. We compared event-free survival between patients who received super-boosted lopinavir and patients who received standard boosted lopinavir, by the Kaplan-Meier method.

Analyses were performed using SPSS software, version 18.0. All tests of significance were two-sided; associations with *P*<0.05 were considered significant.

The study was approved by the Research Ethics Committee at McCord Hospital in Durban, South Africa. The Committee waived the requirement for informed consent for this retrospective data analysis.

## Results

A total of 3025 patients initiated first-line ART at the Sinikithemba clinic at McCord Hospital between July 2004 and February 2007 and over that period 189 (6%) subsequently required second-line LPV/r-containing ART as a result of virologic failure or an adverse effect to initial ART. During that period, we identified 29 patients who received concomitant rifampicin and LPV/r-containing ART, representing 15% of patients who initiated second-line ART. The median age for this subgroup was 36 years (IQR 29–40), 22 patients (76%) were female, the median CD4 cell count and viral load at first-line ART failure was 86 cells/µl (IQR 21–159) and 39,457 copies/mL (IQR 6,025–157,500), respectively. The most common nucleosides used with LPV/r in second-line ART were: AZT + DDI, 11 patients (38%); D4T +3TC, 8 patients (28%); AZT +3TC, 7 patients (24%); 3TC alone, 3 patients (10%).

Lopinavir was super-boosted (LPV/r 400 mg/400 mg twice daily) in 15 (52%) of 29 patients and LPV/r dosing was standard in 14 (48%) of 29 patients (LPV/r 400 mg/100 mg twice daily). We compared the baseline characteristics of patients who received, during tuberculosis therapy, super-boosted LPV/r and the standard dose of LPV/r (Table 1). Among patients receiving super-boosted LPV/r group 93% (14/15) were female and in the standard LPV/r group, 57% (8/14) were female (*P* = 0.03). There were no other significant differences in the baseline characteristics of the two groups at the time of first-line ART failure with respect to age, CD4 cell count, viral load, weight, and second-line nucleoside backbone.

**Table 1:** Baseline characteristics of patients at initiation of lopinavir/ritonavir-based second line ART, according to treatment group.

Characteristic	Lopinavir/ritonavir (400/400 mg)twice daily (n = 15)	Lopinavir/ritonavir (400/100 mg)twice daily (n = 14)	*P* value
Age – yr			
Mean	34.2	37.3	0.45
Range	21–64	11–59	
Female sex – no. (%)	14 (93)	8 (57)	0.03
Weight – kg			
Mean	61.1	54.9	0.26
CD4 cell count at first-line ART failure (cells/ul)			
Median	92	70	0.78
Range	5–203	5–98	
HIV-1 viral load at first-line ART failure (copies/mL)			
Median	33,039	44,926	0.49
Second-line ART nucleoside backbone – no. (%)			
DDI + AZT	6 (40)	5 (36)	
AZT + 3TC	5 (33)	2 (14)	
D4T + 3TC	4 (27)	4 (29)	
Other	0	3 (21)	0.22

**Figure 1:**
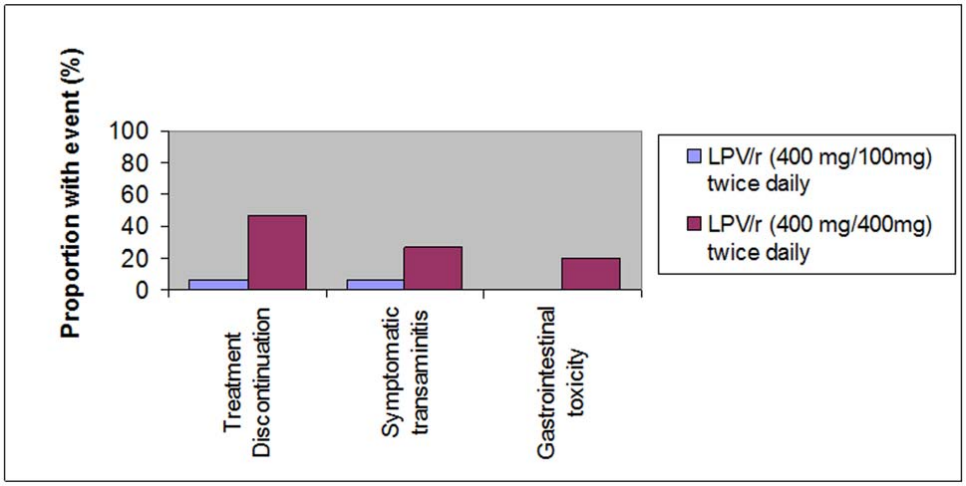
Adverse events among patients who received standard dose and “super-boosted” lopinavir/ritonavir dosing during concomitant treatment for tuberculosis.

Among patients who received coadministered rifampicin-containing TB therapy and LPV/r -based ART, adverse reactions were common. Hepatitis and gastrointestinal toxicity were the most prominent adverse reactions (Figure 1). Overall, ALT elevation of any grade was noted in 5 of 29 (17%) patients; there were no grade 4 ALT elevations or deaths. Patients who received super-boosted ritonavir experienced a trend towards a higher overall rate of symptomatic transaminitis (27% vs. 7%; *P* = 0.3) and gastrointestinal toxicity (20% vs. 0%; *P* = 0.2).

**Figure 2:**
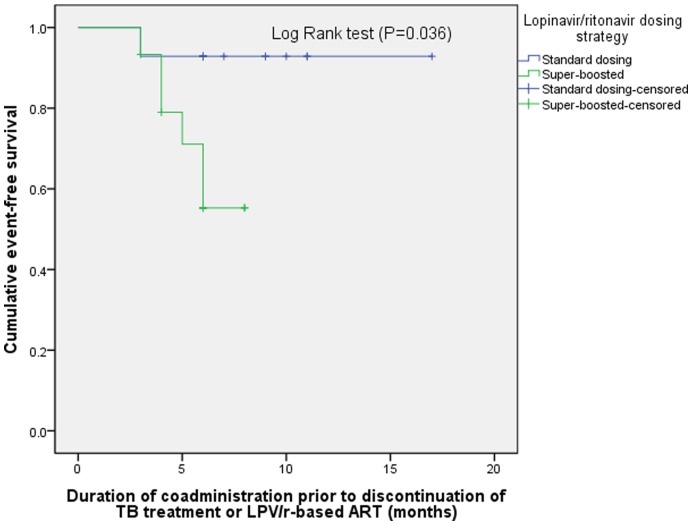
Kaplan-Meier survival curve for the impact of lopinavir/ritonavir dosing strategy among patients with HIV/TB coinfection on time until treatment discontinuation.

Overall, the mean period of coadministration of rifampicin-containing TB therapy and LPV/r-based ART was 6.7 months [range 1–11 months]. Months of coadminstered TB and LPV/r-based ART treatment completed was evaluated as a function of lopinavir/ritonavir dosing strategy utilizing a Kaplan-Meir analysis. Patients who received the super-boosted lopinavir/ritonavir strategy had a significantly fewer months of dual therapy completed prior to treatment discontinuation compared who received standard lopinavir/ritonavir dosing (log rank, *P* = 0.036; Fig. 2). Also, the proportion of patients requiring discontinuation of treatment as a result of an adverse drug reaction was compared between the super-boosted LPV/r and unadjusted-dose groups. Patients who received super-boosted LPV/r were more likely to require treatment discontinuation because of an adverse effect (super-boosted LPV/r, 7 of 15 patients (47%); standard LPV/r, 1 of 14 patients (7%); *P* = 0.035). The most common reason for treatment discontinuation was transaminitis in the presence of clinical symptoms or signs of hepatitis.

To explore if D4T or DDI may have been implicated in higher rates of toxicity among patients receiving co-administered LPV/r and rifampicin-containing TB treatment, we explored the relationship between receiving a nucleoside backbone containing DDI or D4T compared to a backbone without DDI or D4T on rate of symptomatic transaminitis, gastrointestinal toxicity, and rate of treatment discontinuation. There was no significant impact of use of DDI or D4T-containing backbone on rate of symptomatic transaminitis (*P* = 0.775) or rate of gastrointestinal toxicity (*P* = 0.965). A Kaplan-Meir analysis revealed no relationship between use of DDI or D4T in the backbone and treatment discontinuation (log-rank, *P* = 0.606).

We evaluated the proportion of patients in the two ritonavir dose groups in whom virologic failure (HIV-1 RNA >200 c/mL) was observed (Table 2). Overall, virologic failure during coadministration was detected among 7 (24%) of 29 patients. Among 15 patients who received super-boosted ritonavir, 3 (20%) patients experienced virologic failure and among 14 patients who received standard LPV/r, 4 (29%) experienced failure (*P* = 0.7).

**Table 2:** Clinical outcomes associated with coadministration of lopinavir/ritonavir-based ART and rifampicin-containing TB treatment.

Outcome	Lopinavir/ritonavir (400/400 mg)twice daily	Lopinavir/ritonavir (400/100 mg)twice daily
	N = 15	N = 14
HIV outcomes		
Virologic failure (>200 c/mL), number (%)	3 (20)	4 (29)
Tuberculosis treatment outcomes		
Months of overlapping therapy completed (mean)	5.4	8.1 *
Completed	12 (80)	13 (93)
Died or lost to follow-up	3 (20)	1 (7)

T-test, Chi-square, and Fisher’s tests used for comparisons, * p<0.05.

## Discussion

In South Africa, where the prevalence of HIV and tuberculosis are among the highest in the world, we found that, overall, during second-line ART with a LPV/r-containing regimen approximately 15% of patients required coadministration of rifampicin for tuberculosis therapy. In our sample, co-infected patients who received super-boosted LPV/r experienced a trend towards a higher rate of symptomatic transaminitis, gastrointestinal toxicity and the durability of coadministered treatment was significantly shorter resulting from adverse event-related treatment discontinuation (log rank test, *P = *0.036). The most common reason for treatment discontinuation was transaminitis in the presence of clinical symptoms or signs of hepatitis. Early discontinuation of coadministered lopinavir/ritonavir and rifampicin-based TB treatment placed patients at risk for suboptimal treatment outcomes. As the number of patients requiring boosted protease inhibitor-containing therapy for HIV expands, the need for a more varied drug formulary – to respond to common situations such as TB co-infection – grows even more pressing.

Our findings confirmed concerns raised in prior studies about concomitant use of rifampicin with superboosted LPV/r (400 mg/400 mg twice daily) to prevent a reduction in lopinavir plasma concentrations [8], [9]. Currently the use of super-boosting LPV/r as a strategy to overcome the effect of rifampicin on serum lopinavir concentrations in adults is not recommended by the CDC [4]. However our findings contrast with a larger study by Frehoff and colleagues who found – in South African children with HIV and TB coinfection – that super-boosted LPV/r was generally well-tolerated [10]. In the absence of similiar studies in adults, the applicability of the pediatric data to adults with HIV and tuberculosis coinfection is not clear.

We did not find a high rate of virologic failure associated with coadministration LPV/r-based ART and TB treatment, either among patients who received super-boosted LPV/r or standard dose LPV/r, despite the known effect of rifampicin on reducing lopinavir concentrations. However because of our relatively low sample size, we cannot exclude such an effect. It should also be considered that in the current study, patients were not homogenous with respect to time since initiation of second-line ART. Further, it has been previously reported that patients receiving long-term boosted-protease inhibitor-containing ART may tolerate more variation in lopinavir concentrations without loss of virologic control [11].

There are several limitations of this study. First, with regard to the adverse events, we implicated specific drugs (ritonavir and rifampicin) but cannot exclude a role for other drugs including the NRTIs and other anti-tuberculosis drugs in the standard TB treatment regimen including isoniazid. Second, patients who received less than three months of coadministered LPV/r-containing second-line ART and rifampicin-based TB treatment were not included, potentially excluding patients who experienced early adverse events. Third, adverse events were detected passively during coadministration and therefore we are likely to have underestimated the rate of these events. For example, a symptom-based monitoring approach was used to monitor for hepatic toxicity (transaminase levels were measured when symptoms or signs resulted in concern for drug-induced hepatitis), and therefore we were unable to detect subclinical elevations in transaminases. It is possible that the a more intensive lab monitoring strategy would have resulted in more adverse effects detected, but the relevance of subclinical laboratory abnormalities is not clear [12].

Managing the competing risks of coadministered LPV/r-based ART and rifampicin-containing TB treatment is complex, requires access to laboratory services and – with the increasing use of second-line ART – is likely to become a more common problem over time. In the absence of additional drug options, efforts to use existing agents to overcome the interaction between lopinavir and rifampicin have continued. An additional potential dosing strategy was illustrated by a recent small trial involving 18 co-infected patients in South Africa. Decloedt and colleagues used double-dose lopinavir/ritonavir in combination with rifampicin-based TB treatment and found no grade 3 or 4 level toxicity and no evidence of virologic breakthrough [13]. However most evidence suggests that strategies involving increasing the ritonavir dose in adult patients receiving LPV/r is associated with a relatively high rate of adverse events and need for treatment discontinuation. It does not appear to be an optimal long-term solution particularly in the setting of efforts to reduce the need for lab monitoring and the shift management of HIV-infected patients on long-term ART to less trained medical cadres.

The replacement of rifampicin with rifabutin, in patients receiving boosted PIs requiring TB treatment, is recommended in high-income countries. Rifabutin can be substituted for rifampicin in the treatment of TB without loss of efficacy and rifabutin – when given at an adjusted dose of 150 mg every other day – does not substantially lower lopinavir concentrations [14]. However, rifabutin concentrations may not be optimal when given at this adjusted dose, the cost of rifabutin is relatively high and the agent is not widely available in low and middle-income countries [15]. Further rifabutin is not produced in a fixed-dose combination (FDC) making it poorly compatible with TB control programmes which depend upon FDCs rather than individual agents.

More feasible than the wider introduction of rifabutin may be improving access to additional antiretroviral options in resource-poor settings with potentially fewer important interactions with rifampicin-based TB treatment. Raltegravir, an integrase inhibitor which does not require boosting, can be dose-adjusted during rifampicin-based TB treatment, is one promising alternative [16]. However raltegravir is not available as a generic and at current pricing is inaccessible for most countries with generalized HIV epidemics. Support for clinical trials in HIV and TB co-infected patients in resource-limited settings will be critical to determine regiment that will provide effective TB treatment, maintain high levels of virologic suppression and do so with minimal side effects. Until additional therapeutic options are made available in areas with high burdens of HIV and TB – such as South Africa – clinicians will be forced to manage HIV and TB coinfected patients receiving LPV/r-based ART with treatment options that may not be optimal.
