# Correction: Coadministration of Lopinavir/Ritonavir and Rifampicin in HIV and Tuberculosis Co-Infected Adults in South Africa

**DOI:** 10.1371/annotation/730cdfd0-78c5-48fc-a095-f633905ff2f0

**Published:** 2013-12-09

**Authors:** Richard A. Murphy, Vincent C. Marconi, Rajesh T. Gandhi, Daniel R. Kuritzkes, Henry Sunpath

There is an error in the first sentence of the fifth paragraph in the "Methods" section. The correct sentence is: We compared baseline characteristics (at the time of first-line ART failure), rates of key adverse events, treatment discontinuation and virologic suppression among patients receiving second-line ART and concomitant tuberculosis therapy who received super-boosted lopinavir (with ritonavir 400 mg twice daily) and patients who received standard boosted lopinavir (with ritonavir 100 mg twice daily). 

